# Measuring Neighborhood Landscapes: Associations between a Neighborhood’s Landscape Characteristics and Colon Cancer Survival

**DOI:** 10.3390/ijerph18094728

**Published:** 2021-04-29

**Authors:** Daniel Wiese, Antoinette M. Stroup, Aniruddha Maiti, Gerald Harris, Shannon M. Lynch, Slobodan Vucetic, Victor H. Gutierrez-Velez, Kevin A. Henry

**Affiliations:** 1 Department of Geography and Urban Studies, Temple University, Philadelphia, PA 19122, USA; victorhugo@temple.edu (V.H.G.-V.); khenry1@temple.edu (K.A.H.); 2 New Jersey Department of Health, New Jersey State Cancer Registry, Trenton, NJ 08625, USA; nan.stroup@rutgers.edu (A.M.S.); gaharris@cinj.rutgers.edu (G.H.); 3 Rutgers School of Public Health, Rutgers Cancer Institute of New Jersey, New Brunswick, NJ 08901, USA; 4 Department of Computer and Information Sciences, Temple University, Philadelphia, PA 19122, USA; tuf86648@temple.edu (A.M.); vucetic@temple.edu (S.V.); 5 Fox Chase Cancer Center, Division of Cancer Prevention and Control, Philadelphia, PA 19111, USA; shannon.lynch@fccc.edu

**Keywords:** residential histories, residential mobility, time-varying covariates, survival analysis, geographic disparities, colon cancer, neighborhood socio-economic status, landscape metrics, landscape characteristics

## Abstract

Landscape characteristics have been shown to influence health outcomes, but few studies have examined their relationship with cancer survival. We used data from the National Land Cover Database to examine associations between regional-stage colon cancer survival and 27 different landscape metrics. The study population included all adult New Jersey residents diagnosed between 2006 and 2011. Cases were followed until 31 December 2016 (N = 3949). Patient data were derived from the New Jersey State Cancer Registry and were linked to LexisNexis to obtain residential histories. Cox proportional hazard regression was used to estimate hazard ratios (HR) and 95% confidence intervals (CI95) for the different landscape metrics. An increasing proportion of high-intensity developed lands with 80–100% impervious surfaces per cell/pixel was significantly associated with the risk of colon cancer death (HR = 1.006; CI95 = 1.002–1.01) after controlling for neighborhood poverty and other individual-level factors. In contrast, an increase in the aggregation and connectivity of vegetation-dominated low-intensity developed lands with 20–<40% impervious surfaces per cell/pixel was significantly associated with the decrease in risk of death from colon cancer (HR = 0.996; CI95 = 0.992–0.999). Reducing impervious surfaces in residential areas may increase the aesthetic value and provide conditions more advantageous to a healthy lifestyle, such as walking. Further research is needed to understand how these landscape characteristics impact survival.

## 1. Introduction

Neighborhood characteristics can capture social, physical, and economic conditions of the environment in which a person lives [1]. According to Northridge et al. [2], social context and the built environment are two intermediate factors that influence health and well-being at the individual and population level. The social context of a neighborhood is most often defined through its socio-economic and demographic composition using census data (e.g., median income, % living below poverty) [3]. The built environment includes physical characteristics of neighborhoods (i.e., land covers) that are human-made or modified [4], such as buildings, roads, housing conditions, parks sidewalks or greenspace, that can potentially provide the setting for human activity [5].

Therapeutic landscape theory [6] can add depth to our understanding of how landscapes impact health and cancer outcomes [7]. Gesler’s therapeutic landscape theory posits that social and spatial factors are interconnected, and modification of place can have various effects on human health. Moreover, people may have experienced different feelings and healing effects in various places caused by perceptions of landscape aesthetics [6]. Some studies linked the aesthetical value of the landscapes, analyzed through photography of micro-landscapes, to emotional load and restoration [8,9,10,11]. Lee et al. [12] found that neighborhood satisfaction is related to landscape structure. Others suggest that configuration and composition of different types of land cover and land use classes could be influential on place perception and emotions because “perception, cognition, and evaluation are highly interrelated processes” [13], and perception of environmental landscape is important (among others) for movement and social interactions [14,15,16]. In addition, factors such as natural habitat fragmentation and impervious surfaces may also influence health outcomes considering the relation between urban design and mental health [17].

For numerous cancer types, studies report significant associations between neighborhood socio-economic conditions and cancer mortality and survival [18,19,20,21,22,23,24,25,26,27,28,29]. Generally, these studies have found that people living in low socio-economic-status neighborhoods have significantly higher mortality or shorter survival after diagnosis. The relationship is less consistent between the built environment and cancer mortality and survival. Several studies have examined these associations. For example, a study focused on greenspace availability and accessibility by James et al. [30] found that higher levels of green vegetation were associated with decreased cancer mortality. Several other studies on lung cancer-specific mortality did not find any significant associations [31,32,33,34]. For breast cancer, Keegan et al. [35] did not find any positive influence of park availability and survival, but did report an increased risk of breast cancer death in areas with higher traffic/road density, possibly through discouraging recreational-based physical activity.

Among the studies that have examined the relationship between the built environment and non-cancer health outcomes (e.g., cardiovascular and/or mental health illnesses), most have focused on greenspace. Mears et al. [36] summarized positive effects of the urban greenspace on health outcomes, including the reduction of traffic pollution and a reduction of heat-island effects, as well as an increase of emotional recreation effect and physical or social activities. In one study, Bratman et al. [37] examined evidence across the natural, social, and health sciences on the impacts of nature experience on mental health. They argue that the configuration and conception of greenspace is essential for human well-being. Kondo et al. [38] also concluded that navigating through urban greenspaces causes more positive emotions compared to the built urban environment. Additionally, the quality of the greenspace (e.g., well-maintained parks) has a positive influence for psychological well-being [39,40,41,42]. Moreover, urban gardens and well-maintained front yards may influence population health through the pathways of aesthetics and promote walking and outdoor physical activity [43]. Additionally, walkability, mix and type of businesses, and land use composition of a neighborhood may encourage healthy behavior and ‘facilitate integration of habits into a daily lifestyle” (p. 76) [5]. Given the importance of greenspace on health and a lack of studies focused specifically on cancer, more research is needed examining the relationship between the built environment and cancer outcomes in order to better understand these relationships and underlying pathways [44].

With the exception of a few studies [30,35,45], insufficient attention has been given to integration of both social and environmental landscape characteristics on cancer outcomes. Considering that cancer survival has shown to be associated with mental well-being [46,47], integrating landscape and built-environmental characteristics may be useful for quantifying neighborhood quality and help measure the effect of neighborhood land cover configuration and composition on cancer outcomes. 

Estimating the aesthetical value of a landscape and finding the best measure for defining the built environment can be challenging because there are infinite ways to measure and operationalize the data in research [48] and further distil the essential components that impact health and health behaviors. Frequently, the built environment is integrated into modeling using census data such as housing density and quality [44]. However, recent technologies in image classification allow an estimation of neighborhood physical disorder (e.g., presence of abounded buildings, non-maintained roads) or greenspace availability and quality using remote sensing products and street view in health research [49,50,51]. Despite the increasing literature, however, when examining the role of greenspace on health outcomes, several gaps remain, including “standardization” of appropriate measures [38,52]. Therefore, it is important to develop clear concepts and metrics for quantifying and measuring the effects of a neighborhood’s landscape and the built environment on health outcomes to understand the underlying pathways.

Landscape metrics are a commonly used technique in landscape ecology—an environmental science approach of landscape characterization, evaluation, and design [53] to quantify landscape characteristics and features. This concept is frequently applied in urban planning, biodiversity, species richness, and conservation and infectious disease epidemiology studies [54,55]. Landscape metrics are also common in urban aesthetics evaluation [8,9]. To date, there have been no population-based cancer studies that integrate landscape metrics.

In this study, we examine associations between regional-stage colon cancer survival and several landscape metrics that quantify neighborhood’s built-environment using cancer surveillance data from the New Jersey State Cancer Registry, residential histories, and land cover and land use data from the National Land Cover Database.

## 2. Materials and Methods

### 2.1. Study Population

The NJSCR provided all colon cancer cases. The NJSCR is a population-based cancer registry that collects and maintain incidence data for the State of New Jersey. It was established in October 1978 and monitors cancer among the more than 8.9 million residents of New Jersey [56]. The study population includes all New Jersey residents, 18 years and older, with histologically confirmed, first primary regional stage colon cancer, as defined according to the International Classification of Diseases for Oncology, 3rd Edition (ICD-O3 C180–C189, C260; excluding histology codes 9050–9055, 9140, 9590–9992) [57] diagnosed between 1 January 2006 and 31 December 2011 (N = 4041). We restricted our analysis to regional stage colon cancers to simplify the interpretation of the results and reduce systematic variation in survival that could be attributable to stages at diagnosis. The study was reviewed and/or approved by Temple and Rutgers University Institutional Review Boards.

Individual-level factors included age at diagnosis, gender (male, female), race/ethnicity (Non-Hispanic (NH) White, NH Black, NH Asian/Pacific Islander (API), NH Other, and Hispanic (any race)), and vital status, including date of death and cause of death (if deceased) or date of last contact (if alive). Cases were followed until their deaths, relocation from the state of New Jersey, or until 31 December 2016. Deaths attributed to colon cancer were coded based on ICD-10 code C18 [57]. NJSCR regularly updates the database through linkages with state and national sources, including death data from the New Jersey Department of Health Office of Vital Statistics and Registry and the National Death Index, hospital discharge files, Centers for Medicare and Medicaid Services, Social Security Administration Services for Epidemiologic Researchers, and motor vehicle registration files [56]. 

### 2.2. Residential Histories

The NJSCR linked the study population to the commercial residential history database developed by LexisNexis, Inc. (Miamisburg, OH, USA; https://www.lexisnexis.com/en-us/products/public-records.page, accessed on 28 April 2021), similar to earlier studies [58,59,60,61]. The majority of regional stage colon cancer cases [*n* = 3949 (97.6%)] had residential information available for up to 20 of the most recent addresses between 1946 and 2018, with documented start and stop dates. A preprocessing technique described by Wiese et al. [24] was applied to establish a complete residential timeline for the time after diagnosis. All residential addresses were geocoded to the 2010 census tract (CT) boundaries using the North American Association of Central Cancer Registries (NAACCR) AGGIE Geocoder [62]. 

### 2.3. Socio-Economic Variables

The socio-economic variables included a widely used census tract poverty (CT-poverty) variable, defined as the proportion of population 18 years and older living below the Federal poverty level. Additionally, we included information on census tract measures of housing density and median year housing built. The required variables were obtained from publicly available U.S. Census and American Community Survey (ACS) data. U.S. Census 2010 and the ACS 5-year average data 2006–2010, 2007–2011, 2008–2012, 2009–2013, and 2010–2014 were used for residencies between 2006 and 2010. ACS 2011–2015, 2012–2016, and 2013–2017 were used for residencies between 2011 and 2016. 

For each case, all corresponding CTs during the follow-up period were included, and every residential record received a corresponding value based on the earliest date of the residential appearance in the data set. If a patient remained at the same diagnosis CT over multiple years, we assigned annual values to capture changes within the neighborhood that could be caused by the gentrification. 

### 2.4. Environmental Variables

The neighborhood built-environment factors were measured by using landscape metrics that captured characteristics such as land cover and land use composition and configuration of the neighborhood considering local spatial patterns. 

The National Land Cover Database (NLCD) was used to extract land cover classes and proportion of imperviousness for the years 2006 (used for residential records 2006–2009), 2011 (used for residential records 2010–2014), and 2016 (used for residential records 2015–2016). The NLCD products are freely available raster files of 30 m spatial resolution that were classified using Landsat-based satellite imagery by the U.S. geological survey [63]. The land cover raster includes 16 categorical classes and the imperviousness raster, which has a continuous scale of proportion of impervious surfaces per pixel. To reduce the number of categories for further analysis, we reclassified the original NLCD raster using R package raster [64] into 7 classes (Forest, Grass, Shrubs, Developed Lands (Open, Low-, Medium-, and High-Intensity) (Table 1). Land use information was obtained from the New Jersey Geographic Information Network (njgin.nj.gov/njgin/edata/parcels/#!/, accessed on 28 April 2021), which includes publicly available information on parcel use for the state of New Jersey as of the year 2019. The original shapefile (spatial polygon) was rasterized (i.e., converted) using R package raster [64] based on the parcel use category and reclassified by keeping only industrial and commercial lands.

Previous studies have already examined the NLCD’s classification of developed lands to estimate greenspace availability in urban areas and have noted that open and low-intensity developed lands are suitable for identification of greenspaces and trees [34,65,66,67]. Typically, areas with predominantly large housing parcels, roads surrounded by greenspaces, urban housing with larger backyards, or isolated large roads would be classified as developed open land (NLCD class 21), assuming a larger proportion of greenspaces than of impervious/built surfaces (max. 20%) in the area. In contrast, a central business district, densely built inner-city housing, or a largely expanded shopping mall would be classified as high-intensity developed lands (NLCD class 24) with 80–100% imperviousness.

**Figure 1:**
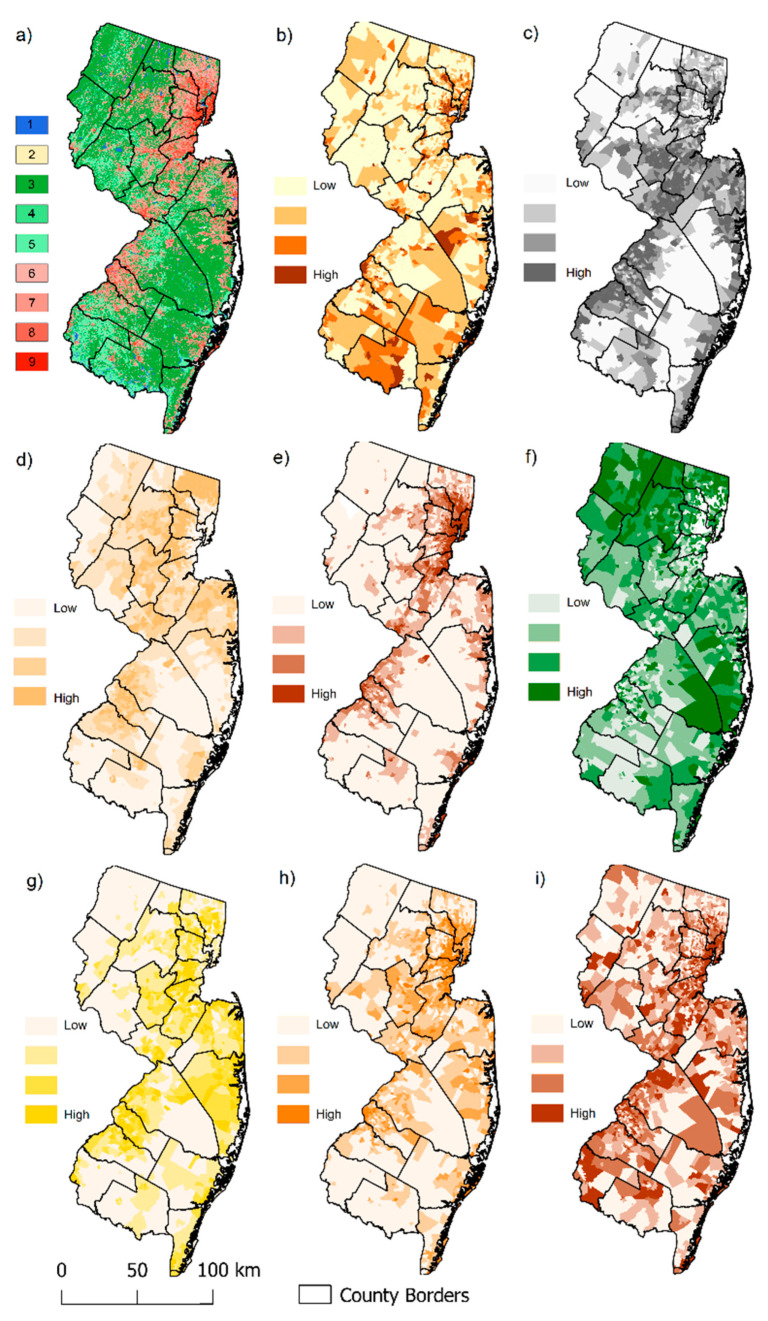
Land cover classification and landscape characteristics of New Jersey: (**a**) Reclassified land cover classification (1 = Water, 2 = Sand/Bare, 3 = Forest, 4 = Shrubs, 5 = Grass, 6 = Open, 7 = Low, 8 = Medium, 9 = High intensity developed lands); (**b**) Census tract poverty level by categories (0–5%, 5–10%, 10–20%, 20+%); (**c**) Shannon Diversity Index (SHDI) by quartiles low to high; (**d**) Proportion of open developed lands by quartiles low to high; (**e**) Proportion of high-intensity developed lands by quartiles, low to high; (**f**) Forest contiguity index by quartiles, low to high; (**g**) Aggregation index (AI) of open developed lands by quartiles, low to high; (**h**) Aggregation index (AI) of medium-intensity developed lands by quartiles, low to high; (**i**) Aggregation index (AI) of high-intensity developed lands by quartiles, low to high.

Landscape metrics on patch (square) and class levels (greenspaces) within a landscape (i.e., census tract) were calculated using R statistical software [68] (version 4.0.1), implementing packages landscapemetrics [69] and SDMTools [70]. Landscape metrics include more than 50 measures. Because most variables are highly correlated or difficult to interpret and compare between landscapes because of the open, non-fixed range scale [53,71], we developed a list of the influential landscape metrics, as defined by previous studies [36,39,42]. Table 2 summarizes all landscape metrics that were considered in the present study. Selected landscape metrics and the land cover classification were also mapped using QGIS v.3.10 (Figure 1).

### 2.5. Statistical Analysis

The survival time for every patient was calculated in months as the difference between the date of diagnosis and the date of death or date of last contact. Cases missing survival time (i.e., only ascertained through death certificates or autopsy) were excluded from this analysis. Patients were censored at the date of death if they died from causes other than colon cancer, the date the patient was lost to follow-up, at the end of the follow-up period (31 December 2016), or at the time of the relocation from the State of New Jersey, whichever occurred first. Additionally, we calculated time intervals to every CT location and assigned start and end dates based on residential histories. Every time interval received a corresponding socio-economic and environmental neighborhood value, as well as the vital status of the patient (1-dead, 0-alive).

**Figure 2:**
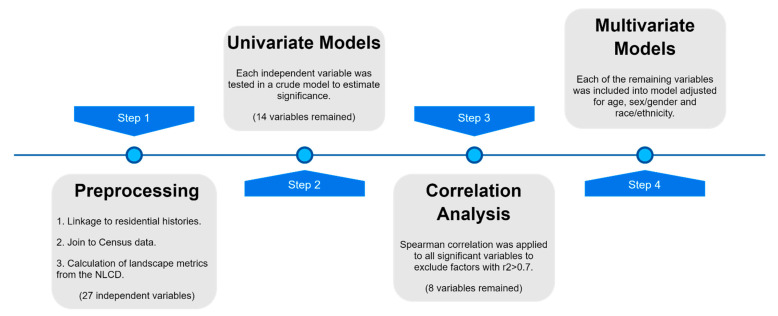
Workflow diagram.

We designed a process for variable selection and evaluation with minor modifications based on methodology from a previous neighborhood wide association study or NWAS [80]. All methodological steps are summarized in Figure 2. First, we developed a series of univariate, crude models to estimate the effect of each selected landscape metric and other neighborhood variables on the duration of survival time using Cox proportional hazard regression for time-varying covariates [81]. Cox proportional hazard regression is a widely used semi-parametric time-to-event modeling technique, where death is considered being the event. Cox proportional hazard regression allows incorporation of individual and area-level covariates. Additionally, it does not require the definition of a probability distribution in advance, and is suitable for time-varying covariates [82]. Variables were selected if they reached significance at *p* < 0.05.

**Figure 3:**
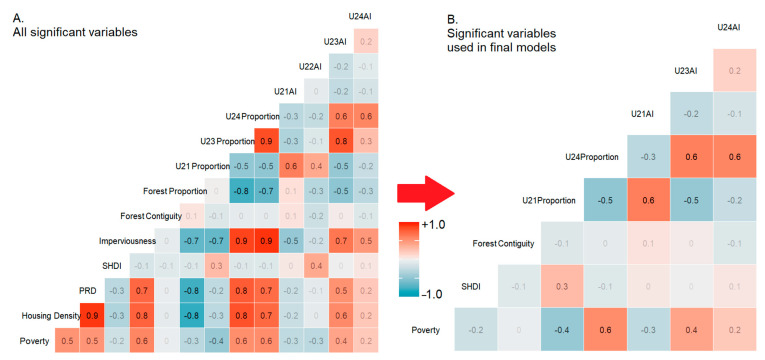
Correlation matrix before and after reduction of significant variables: (**A**) All significant variables based on the univariate models; (**B**) Reduced set of significant variables used in final multivariate models. (Note: Land cover classes are based on the reclassified NLCD raster: U21 = Open, U22 = Low, U23 = Medium, U24 = High intensity developed lands). AI = Aggregation Index, SHDI = Shannon Diversity Index, CI = Contiguity Index, PRD = Patch Richness Density.

Then we applied Spearman correlation analysis of the selected variables because many landscape metrics are highly correlated and redundant. After excluding all highly correlated variables (r^2^ > 0.7), the number of variables was reduced from 14 to 8 (Figure 3). The remaining variables included aggregation indices of all four intensity levels of developed lands, proportion of high intensity developed lands, forest contiguity index, Shannon diversity index, the patch richness density, and CT-poverty (Figure 1).

We then developed a set of models that included all individual-level variables (sex, age and race/ethnicity, sub-stage, and mover status) and each neighborhood variable or landscape metric. To estimate the risk of death from colon cancer by each individual and area-based variable, all coefficients were exponentiated and expressed as Hazard Ratios (HRs). For continuous variables, positive HRs indicate a positive association with the increase in risk of death. For categorical variables, the HRs are compared to the reference group. An HR = 1 indicates that the risk is similar across groups [83]. 

All models were run using R package survival [84] and survsim [81], and met the proportional hazard assumption based on the examination of Schoenfeld residuals using the cox.zph() function in the R survsim [81]. 

## 3. Results

### 3.1. Study Population

The study population included 3949 regional stage colon cancer cases. The spatially interpolated distribution of cases and total population density are presented in Figure S1. There were slightly fewer males (47.6%) than females (52.4%), and about three quarters (73.6%) were NH-White, 12.4% NH-Black, 8.1% Hispanic origin (any race), 3.6% NH-API, and 2.4% Other race. Approximately a third (27.5%) of all the patients died from the colon cancer by the end of follow-up, with a median survival of 66 months (range 1–139). During the follow-up period, 65.5% remained at their diagnosis CT, and 12.1% left New Jersey during the study period. Among those who moved, 18.5% only moved once, 12.3% moved twice, and 3.6% moved three or more times after cancer diagnosis. The average time spent at the CT at diagnosis was 7.5 years (Table 3).

**Figure 4:**
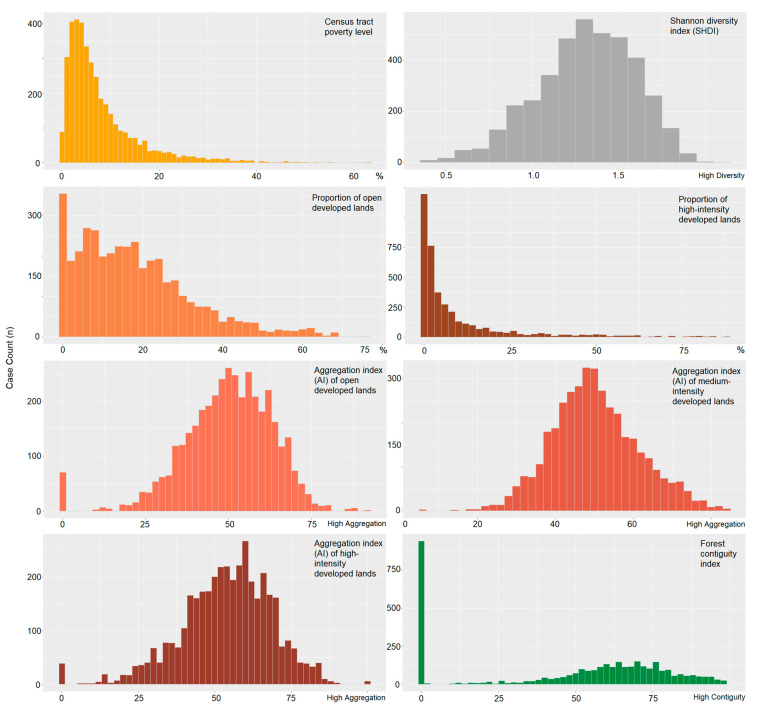
Distribution of colon cancer patients by neighborhood/landscape characteristics.

The distribution of colon cancer patients by neighborhood/landscape characteristics at the time of diagnosis showed that the majority of patients (73.9%, *n* = 2919) lived in areas with a poverty level of less than 10%. Additionally, most patients (82.5%, *n* = 3259) were living in neighborhoods with a relatively low (≤30%) proportion of open developed lands (areas with large greenspace cover), while 89.8% (*n* = 3547) of patients living in neighborhoods where more than 30% of the total landscape was dominated by high-intensity developed lands (less than 20% greenspaces). Twenty-four percent (*n* = 939) of all colon cancer patients were residents in a neighborhood with no tree cover. The distribution of the study population for these and other landscape metrics are summarized in Figure 4.

### 3.2. Univariate Models

**Figure 5:**
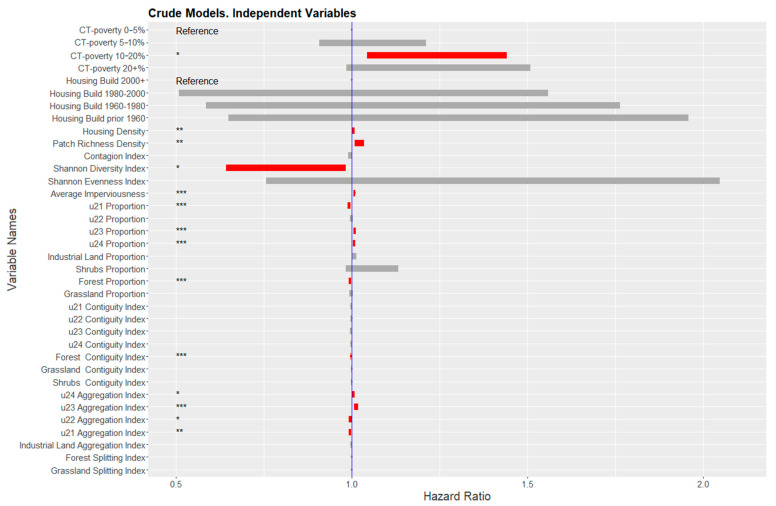
Hazard Ratios of neighborhood-level variables from the crude models, adjusted for each variable individually. Blue line indicates there is no difference in risk. Red bars indicate the 95% confidence interval of statistically significant variables. (Note: Significance levels <0.05 = *; <0.01 = **; <0.001 = *** Land cover classes are based on the reclassified NLCD raster. U21 = Open, U22 = Low, U23 = Medium, U24 = High intensity developed lands).

Figure 5 summarizes the model results for each variable. For every 10% increase in housing density, the risk of death increased by 5% (HR = 1.005; 95% CI = 1.002–1.009). For CT-poverty, cases living in CT with poverty levels in the range of 10–20% had a higher risk of death than those living in CTs with poverty levels in the range of 0–5% (HR = 1.23; 95% CI = 1.04–1.44).

Of the 27 landscape metrics, six were statistically significant. An increasing proportion of the high- and medium-intensity developed lands were positively associated with the risk of death (HR = 1.007; 95% CI = 1.003–1.01 and HR = 1.008; 95% CI = 1.005–1.011, respectively). Similarly, a positive association was found between an increasing aggregation index of high- and medium-intensity developed lands and risk of death (HR = 1.005; 95% CI = 1.001–1.009; HR = 1.01; 95% CI = 1.007–1.02, respectively). There was also a positive association between increasing patch richness density (HR = 1.02; 95% CI = 1.01–1.03) and elevated risk of death. Average imperviousness was also significant, indicating an approximate 7% risk increase for every 10% increase of the neighborhood’s imperviousness (HR = 1.007; 95% CI = 1.004–1.011).

Seven landscape metrics were significantly associated with a decrease in risk of colon cancer death, including aggregation of low-intensity developed lands (HR = 0.996; 95% CI = 0.991–0.999), proportion and aggregation of open developed lands (HR = 0.992; 95% CI = 0.988–0.997, HR = 0.996; 95% CI = 0.991–0.999, respectively), Shannon diversity index (HR = 0.79; 95% CI = 0.64–0.98), and proportion and contiguity (i.e., connectivity) of forest/trees (HR = 0.995; 95% CI = 0.992–0.998, HR = 0.997; 95% CI = 0.996–0.998, respectively).

### 3.3. Multivariate Models

In the last step, we developed ten multivariate models, adjusting for individual level variables (age and sub-stage, sex/gender, race/ethnicity, and mover status), CT-poverty, and each landscape metric. The individual level HRs are provided in Supplementary Table S1.

**Figure 6:**
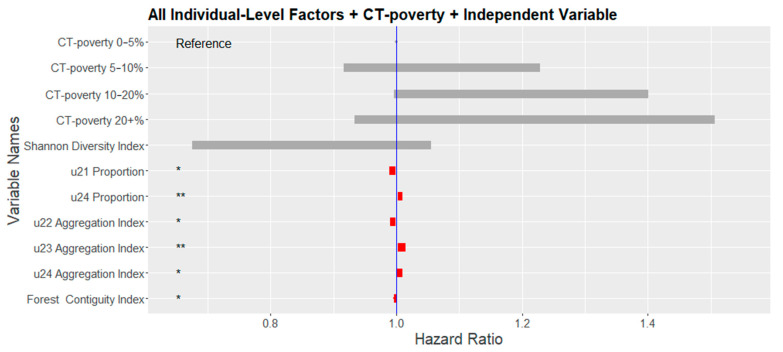
Hazard Ratios of neighborhood-level variables from the multivariate model adjusted for all individual-level factors and each neighborhood/landscape variable individually. Blue line indicates a zero-coefficient or no difference in risk. Red bars indicate the 95% confidence interval of statistically significant variables. (Note: Significance levels <0.05 = *; <0.01 = **; Land cover classes are based on the reclassified NLCD raster. U21 = Open, U22 = Low, U23 = Medium, U24 = High intensity developed lands).

In multivariate models, the proportion of high-intensity developed lands had the strongest association with the colon cancer risk of death. For every 10% increase in the proportion, the risk of death increased by 6% (HR = 1.006; 95% CI = 1.002–1.01). Additionally, increasing aggregation index (i.e., more compact areas) of high-intensity developed lands was positively associated with the risk of colon cancer death (HR = 1.005; 95% CI = 1.001–1.009). Similar associations were found for the increasing aggregation index of medium intensity developed lands (HR = 1.009; 95% CI = 1.003–1.015). In contrast, a 6% decrease in risk of colon cancer death was estimated for every 10% increase in the proportion of open developed lands (HR = 0.994; 95% CI = 0.988–0.999). Increasing aggregation index of low-intensity developed lands (HR = 0.995; 95% CI = 0.99–0.999) and increasing forest contiguity index (HR = 0.998; 95% CI = 0.996–0.999) were all significantly negatively associated with the risk of colon cancer death. CT-poverty was no longer significant in multivariable models (Figure 6).

## 4. Discussion

To date, only a few studies have integrated landscape characteristics into cancer disparities research. Traditionally, socio-epidemiological neighborhood studies focus on examining associations between neighborhood factors and cancer survival using Census-based socio-economic status data. However, the physical environment [85], recreational activities [86], and urban design are important components in the selection of residence, especially among older adults [87]. While experiences of place are recognized and there have been several attempts to incorporate the role of the landscape characteristics into cancer research [35,44], often it is done through the utilization of road networks or based on parcel use, greenspace availability, and accessibility. Only few attempts were made using satellite imagery-based products [45,88,89]. 

We evaluated the relationship between the risk of death from colon cancer and several area-based landscape characteristics that attempt to describe the configuration and composition of the built environment after adjusting for individual-level factors and CT-poverty. This study adds to a body of literature on the effects of neighborhood landscape characteristics on cancer survival. Independent of CT-poverty and several individual demographic and prognostic factors, we found a significant relationship between the risk of death from colon cancer and the proportion and aggregation of high-intensity developed lands (i.e., areas dominated by buildings and roads). The risk of death increased as the proportion and aggregation of high-intensity developed lands increased. The reasons for this relationship may be attributed to a low prevalence of greenspaces in areas with more high-intensity developed lands. Less greenspace in these places could reduce access to recreational sites that promote physical activity. Furthermore, such places may evoke negative emotional feelings and psychological well-being that have been found to be associated with a lack of greenspace [37]. Creating landscapes that promote exercise and active transport such as biking and walking is particularly important to cancer patients. Many clinical trials have shown the positive effects that walking and physical activity can have on quality of life and survival time in cancer patients [90], and are known to reduce risk of colorectal cancer development [91,92].

The relationship of worse survival with increasing high-intensity developed lands independent of CT-poverty is likely related to what this metric is capturing. High-intensity developed lands are characterized by places that include less than 20% greenspace of total land area [63]. An increasing proportion and aggregation of high-intensity developed lands is generally characterized by very compact and large areas within neighborhoods, with few or no parks and where the majority of greenspace is a result of overgrown vegetation from abandoned lots. A study from Philadelphia (PA, USA) reported that abandoned buildings and lots were associated with negative health outcomes among residents because of dangerous physical and social environments and sanitation/garbage issues [93]. High-intensity developed lands are also characterized by large parking lots and multiple lane roads that can reduce the aesthetic value of a neighborhood. This can lead to a reduction in walkability. Several previous studies reported the importance of aesthetic value and quality of the neighborhood environment on mental health and physical activity [36,37,42,43,52,65,94]. Additionally, we cannot exclude that this finding could also result from unmeasured confounding factors such as environmental pollution in highly urbanized areas or food deserts.

The better survival in neighborhoods with a higher proportion of open developed lands is likely a result of the large and compact greenspaces found in these areas, which provide potential for recreational activities. Larger open developed lands also assume more contiguous areas with a large proportion of street tree canopy, urban gardens, or large backyards across the landscape (neighborhood). Having vegetation along the roads, green front and backyards could have positive emotional effects [36,43] essential for many cancer survivors. Kondo et al. [38] also conclude that navigating through urban greenspaces compared to built urban spaces leads to more positive emotions. Additionally, this could suggest that the availability of green infrastructure and neighborhood parks would increase walkability and exercising [43,95,96]. 

We also found that increasing the forest contiguity index was positively associated with a decrease in the risk of death. This suggests that lower shape complexity and lower interspersion of greenspaces (i.e., larger and better-connected greenspaces) may further decrease the risk of colon cancer death because of the availability of green corridors within cities. Large contiguous green space leads to more varied use and extended use for physical activity and provides an opportunity to strengthen social capital that improves survival. This aligns with an earlier study reporting a positive effect on general and mental health from having fewer, but larger patches/areas of greenspaces, rather than a high density of small patches [39]. This finding confirms our earlier hypothesis that lack of greenspaces in neighborhoods negatively influences colon cancer patients. As Mary Soderstrom [97] argues, increasing street greenery and number of greenspaces can make a difference and create a dense and pleasurable city at the same time. 

While access to and availability of greenspace is recognized as an (predominantly positive) influential factor on health conditions, the reduction of impervious surfaces and spaces occupied by oversized residential-area roads and gigantic parking lots might be as important as the creation of green infrastructure through zoning or the re-use of abandoned lots. Unfortunately, this practice is rare in the U.S., but more attention must be given to the aesthetics of the neighborhoods. Therefore, we agree with Kondo et al. [38] that “urban planners and public health professionals need evidence of the impacts of specific therapeutic or place-based interventions to help address public health issues facing their constituents” (p. 22) and argue that integration of landscape metrics into health disparities research may provide the required evidence.

Remote sensing-based classification is a valuable tool for land cover analysis and can be customized depending on the research question and study area. Additionally, there is a growing amount of data on a high spatial resolution allowing fine classifications. However, the land cover classification schema developed by the NLCD is informative for analyzing urban and urbanized areas, and offers enormous opportunities for integration of land cover data into cancer research also on nationwide scale, including non-contiguous states and Puerto Rico [63]. Additionally, it is available for several time points, which allows temporal analysis and application of longitudinal study design like in our example. On the other hand, the calculation of various landscape metrics allows a straightforward integration of several measures of landscape characteristics. These could then be helpful for city planning and the establishment of specific cancer prevention and control strategies. 

Moreover, while aesthetical value of a neighborhood is a very subjective measure and typically requires qualitative interviews or surveys, some landscape metrics offer an opportunity for quantification. The contiguity or aggregation indices used here may provide information about landscape configuration [53,71] and become an alternative to more complicated measures derived from Google street view, which are time-consuming and more expensive to process.

The present study has several limitations. While we found evidence for associations between the land cover configuration and risk of death from colon cancer, the results may not be generalizable to other states with different demographic, socio-economic, or landscape characteristics because the study population was limited to New Jersey only. Additionally, we did not have access to individual-level factors such as individual socio-economic status, general health conditions (e.g., obesity data) and behavior. Not only could these factors potentially confound the relationship between landscape characteristics and colon cancer survival, but accounting for individual-level factors may reduce the geographic variance explained by neighborhood and landscape characteristics [98].

Another limitation is that we restricted our study population to regional-stage colon cancer cases and followed patients for only 10 years after diagnosis. This was necessary to minimize extreme variations in survival and limit sources of variation in residential history measures. Therefore, analyzing colon cancer cases diagnosed in earlier (local) or later (metastatic) stages of the disease may result in a different conclusion.

Moreover, we utilized only residential histories collected from LexisNexis. We did not have access to self-reported information and could not validate and/or augment LexisNexis data. However, according to previous studies, the concordance between LexisNexis addresses and addresses collected from study participants (85–86%) is high [58,59]. In our study, we could only validate the residential location at the time of diagnosis between the LexisNexis and the NJSCR. The concordance rate was approximately 83%. Opening to a 6-month window before and after the diagnosis date substantially increased the concordance rate to 93%. Only 8% of the locations from LexisNexis cases did not match any locations reported by the NJSCR. Several factors such as incorrect links at LexisNexis, incorrect geocodes assigned by NJSCR for both registry and LexisNexis residential addresses, incorrect addresses reported to the registry by hospitals and other reporting facilities or geocodes assigned to addresses based on post office boxes could be the reason for address discordance. However, the extent of the bias in either direction would be minimal because of the low proportion of affected cases. In contrast to the residential histories from self-reported data, LexisNexis is an objective source of residential history data, and is not sensitive to potential recall bias. 

Additionally, the application of landscape metrics in neighborhood research is not typical and is more common in natural landscapes for ecological analysis and modeling. In this study, we used census tract boundaries for defining neighborhoods or landscapes. The definition of a landscape as a census tract is a subject of modified area unit problem. The selection of the landscape boundaries is essential for calculating landscape metrics, and change in area size and shape will affect the values of multiple indices. Defining landscapes through a use of other administrative boundaries or grid system may result in different conclusions. However, census tract is a common unit of analysis in public health research and allows an uncomplicated merge of data from various sources. 

Lastly, the spatial resolution of the land cover classification raster was 30 m and suggests that all features within an area of 900 m^2^ are generalized and defined as one class. The establishment of land cover classification with a fine spatial resolution would result in higher precision in the classification of the ground objects. However, 30 m spatial resolution is widely used in remote sensing discipline for land cover classification, and the utilized NLCD dataset is a well-known high-quality product. 

The calculation of landscape metrics can be done on any spatial resolution, but values may vary with change in pixel size. More challenging is the selection of landscape metrics itself. There are many measures on various geographic levels. Thereby, most metrics are highly correlated and redundant. Previous research in landscape ecology suggests that a minimal number of metrics (e.g., number of patch types, mean edge/area ratio, contagion, average patch shape, fractal measurements) would be sufficient to quantify spatial heterogeneity [71,72,73]. However, in a public health context, the selection of landscape metrics should be done more carefully, selecting meaningful variables that can be easily translated to the policy makers and urban planners. Selection of other landscape metrics could result in different associations or cause complications in interpretation.

## 5. Conclusions

The associations between neighborhood socio-economic status and cancer survival are fairly established, where increased risk of death is often associated with high neighborhood poverty. However, the neighborhood environment is not limited to socio-demographic factors because buildings, roads, greenspaces, and other human-made objects dominate landscapes, especially in urban and urbanized areas. It is essential to understand the relationship between landscape characteristics and health outcomes to develop new policies in urban planning and design essential for population health, especially in urban and urbanized areas. Our results suggest that increasing proportions and connectivity of urban greenspaces may substantially decrease the risk of colon cancer death. This association did not change, even after adjusting for neighborhood poverty, which is reported to be associated with a lack of greenspaces in urban and suburban areas [99], and reflected in our correlation analysis. The integration of remote sensing-based products, the NLCD and the calculation of landscape metrics allow the exploration of undiscovered pathways between neighborhood characteristics and colon cancer survival and should be further evaluated in neighborhood studies with other cancer sites and outcomes such as stage at the diagnosis. Additionally, further research is needed to understand how these specific landscape characteristics impact survival, and evaluate opportunities for developing a socio-environmental deprivation index combing census-based variables and land cover metrics in order to identify neighborhoods in need of interventions. Moreover, future studies should include additional neighborhood variables, especially related to walkability, that could help to evaluate the association between neighborhood built environment and colon cancer survival.

## Figures and Tables

**Table 1:** Overview of the land cover classes before and after the reclassification of the NLCD dataset.

The NLCD Code	Reclassification	Commentary
21: Developed Open Space	U21: Developed Open Space	Max 20% imperviousness per cell/pixel
22: Developed Low Intensity	U22: Developed Low Intensity	20–49% imperviousness per cell/pixel
23: Developed Medium Intensity	U23: Developed Medium Intensity	50–79% imperviousness per cell/pixel
24: Developed High Intensity	U24: Developed High Intensity	80–100% imperviousness per cell/pixel
41: Deciduous Forest	Forest	Dominated by tree canopy and includes any type of parks and squares
42: Evergreen Forest		
43: Mixed Forest		
52: Shrub/Scrub	Shrubs	Dominated by shrubs; present on empty housing parcels
71: Grasslands/Herbaceous	Grassland	In urban areas, may assume a low-quality green space
81: Pasture/Hay		
82: Cultivated Crops		
90: Woody Wetland	Forest	Woody wetlands are common in southern New Jersey and have large proportions of deciduous trees
95: Emergent Herbaceous Wetland	Grassland	Herbaceous (also grassy) wetlands are typical for many coastal regions.

**Table 2:** Overview of the area-based and individual variables.

Variables	Land Covers	Definition	Commentary
**Land Cover Class-Level Metrics**			
Class Proportion	Forest, Grass, Shrubs, Industrial, Developed Lands (Open, Low, Medium, High Intensities)	Composition metric. Proportional coverage—% of the landscape covered by each type.	Used by [72,73] and recommended by [36] for green and water space; [74] recommended for Forest, Shrubs and Grass
Aggregation Index (AI)	Developed Lands (Open, Low, Medium, High Intensities), Industrial Areas	Configuration metric. Computed as an area-weighted mean class aggregation index, where each class is weighted by its proportional area in the landscape.	Redundant with several other metrics of proportion, cohesion, and contiguity and may be a meaningful alternative [53]
Splitting Index	Forest, Grass, Shrubs	Configuration metric. A large splitting index, results from land covers being split into many patches with an even size distribution.	Correlated with the aggregation index. Applied for green spaces only in relation to health outcomes [39].
Contiguity Index (CI)	Developed Lands (Open, Low, Medium, High Intensities), Forest, Grass	Configuration metric. Large contiguous patches will result in larger contiguity index values.	CI for green/tree land cover classes associated with health outcomes [39].
**Landscape-Level Metrics**			
Shannon Diversity Index	Based on all Land Cover Classes	Composition metric. The more classes and the more equally distributed, the higher the index.	Used for measuring the aesthetic value and diversity [8]. Associated with health outcomes [39].
Patch Richness Density (PRD)	Based on all Land Cover Classes	Number of patches per hectare. High values indicate high dispersion	PRD for green areas and recreational lands associated with poor health [39].
Contagion Index	Based on all Land Cover Classes	Composition metric. High values indicate result from landscapes with a few large, contiguous patches and low dispersion and interspersion of patch types	
Average Proportion of Imperviousness	Census Tract Average based on NLCD dataset estimating imperviousness proportion per pixel	Composition metric. Highly negatively correlated with Tree Canopy proportions but is more accurate	Highly correlated (negative) with Tree Canopy Cover but more accurate [75,76]
**Census-Based Variables**			
Poverty Level by Category	Percentage of population 18 and older living below federal poverty level.	Socio-economic status	Associated with cancer outcomes including survival and mortality
Median Year Structures Built	Median that the areas residential buildings were constructed.	Organized into categories.	Housing age and conditions are associated with health outcomes [17,77] and poverty [78]
Housing Density	Number of structures per area unit (acre)	Continuous variable defined by census tract	Potential intermediate factor in health outcomes [79]

**Table 3:** Study population characteristics.

	Overall (*n* = 3949)
**Age**	
Mean (SD)	65.8 (13.3)
Median [Min, Max]	68.0 [21.0, 85.0]
**Gender**	
Male	1878 (47.6%)
Female	2071 (52.4%)
**Race/Ethnicity**	
NH-White	2902 (73.5%)
NH-Black	488 (12.3%)
Hispanic (any race)	325 (8.2%)
NH-API	141 (3.6%)
Other	93 (2.4%)
**Regional Stage Subcategory**	
Regional, direct extension only	1339 (33.9%)
Regional, lymph nodes only	1268 (32.1%)
Regional, both	1342 (34.0%)
**Vital Status**	
Censored	2862 (72.5%)
Colon Cancer Death	1087 (27.5%)
**Survival Time (months)**	
Mean (SD)	62.3 (38.0)
Median [Min, Max]	66.0 [1.00, 139]
**CT Changes (Type of “moves”)**	
CT at Date of Diagnosis Only	2587 (65.5%)
Change in Residential CT within NJ	885 (22.4%)
Change in Residential CT outside NJ	477 (12.1%)

## Data Availability

Due to the nature of this research, supporting patient data is not available.

## Supplementary Materials

The following are available online at https://www.mdpi.com/article/10.3390/ijerph18094728/s1, Table S1: Hazard Rates of individual-level factors from the multivariate model. Figure S1: Distribution of population density by municipality and interpolated density of colon cancer patients’ residencies.
