# Supplementary material for: Measuring Neighborhood Landscapes: Associations between a Neighborhood’s Landscape Characteristics and Colon Cancer Survival
Source: Int J Environ Res Public Health. 2021 Apr 29;18(9):4728. doi: 10.3390/ijerph18094728 (PMC8124655; doi:10.3390/ijerph18094728)
Supplement: Supplementary file 1 [file ijerph-18-04728-s001.zip › ijerph-1178402-supplementary.pdf]

Supplementary Files

Table S1: Hazard Rates of individual-level factors from the multivariate model.

Individual-Level Factors	Coefficient	Hazard Rate (95% Confidence Interval)	P-Value
Male	Reference		
Female	-0.21	0.81 (0.73-0.9)	<0.001
Age at diagnosis	0.02	1.025 (1.02-1.03)	<0.001
Regional. direct extensions only	Reference		
Regional. lymph nodes only	-0.39	0.68 (0.58-0.78)	<0.001
Regional. direct extension and lymph nodes	0.6	1.83 (1.62-2.07)	<0.001
NH-White	Reference		
NH-Black	0.45	1.56 (1.35-1.81)	<0.001
Hispanic	0.49	1.64 (1.37-1.96)	<0.001
Asian/Pacific Islanders	0.2	1.22 (0.92-1.62)	0.17
Others	0.13	1.14 (0.79-1.64)	0.45
Non-Movers	Reference		
Movers	1.15	3.17 (2.84-3.53)	<0.001

Note: Hazard Rates and 95% confidence intervals are exponentiated the parameter estimates/coefficients

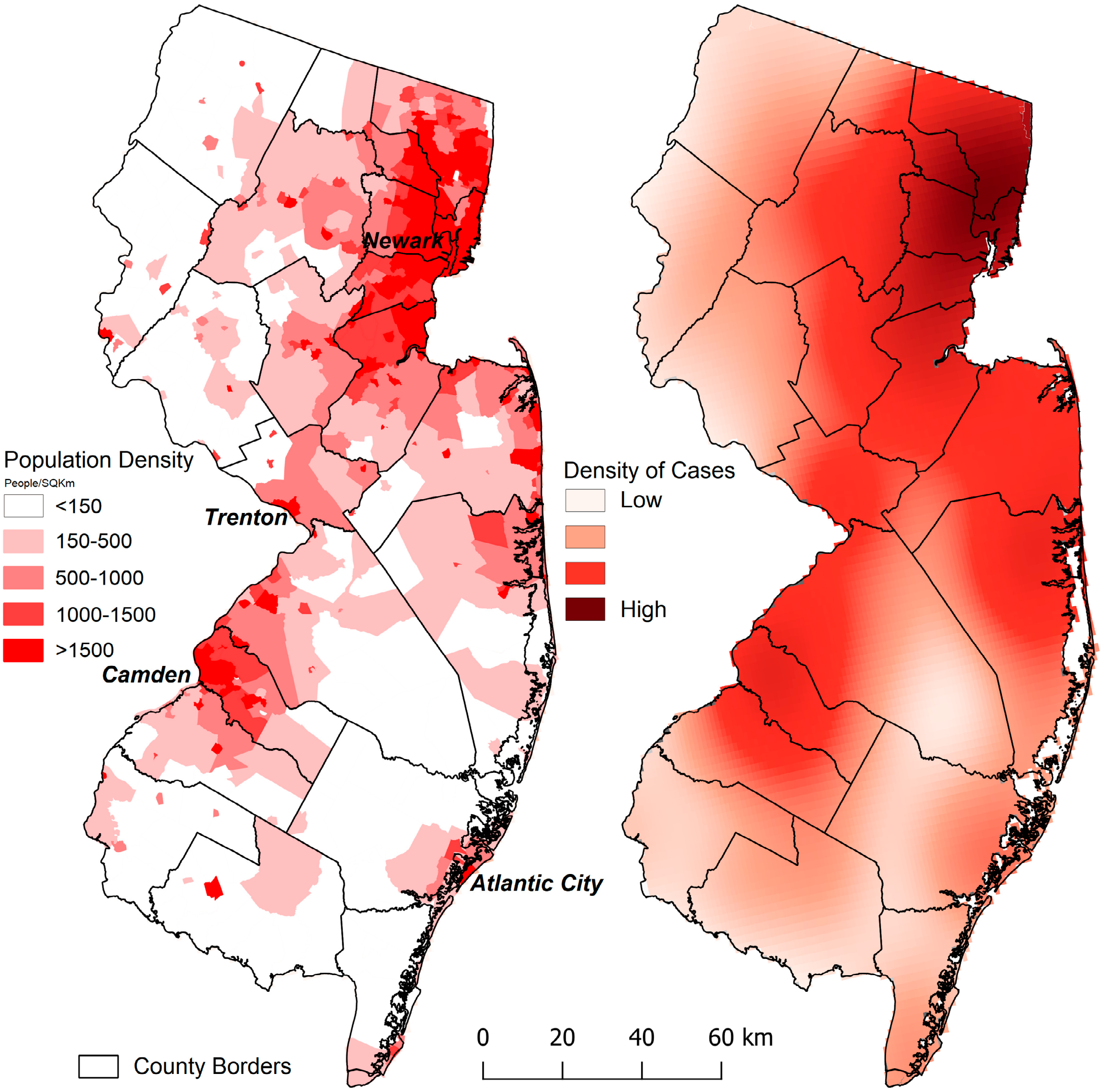

Figure S1: Distribution of population density by municipality and interpolated density of colon cancer patients' residencies. The geographic distribution of patients reflects the statewide population density patterns.

Note: Density of Cases was interpolated on a spatial 1 km grid to keep confidentiality.
